# Antinociceptive and Anti-Inflammatory Effects of Ethanol Extract from *Vernonia polyanthes* Leaves in Rodents

**DOI:** 10.3390/ijms13033887

**Published:** 2012-03-22

**Authors:** Vanessa dos Santos Temponi, Jucélia Barbosa da Silva, Maria Silvana Alves, Antônia Ribeiro, José de Jesus Ribeiro Gomes de Pinho, Célia Hitomi Yamamoto, Miriam Aparecida Oliveira Pinto, Glauciemar Del-Vechio-Vieira, Orlando Vieira de Sousa

**Affiliations:** 1 Graduate Program in Pharmaceutical Sciences, Faculty of Pharmacy, Federal University of Juiz de Fora, Campus Universitário, Juiz de Fora, Minas Gerais 36036-330, Brazil; E-Mails: vanessatemponi@hotmail.com (V.S.T.); juceliabs@yahoo.com.br (J.B.S.); 2 Department of Pharmaceutical Sciences, Faculty of Pharmacy, Federal University of Juiz de Fora, Campus Universitário, Juiz de Fora, Minas Gerais 36036-330, Brazil; E-Mails: alves_ms2005@yahoo.com.br (M.S.A.); jose.pinho@ufjf.edu.br (J.J.R.G.P.); hytomani@yahoo.com (C.H.Y.); miriamaop@yahoo.com.br (M.A.O.P.); glauciemar@gmail.com (G.D.-V.-V.); 3 Department of Biochemistry, Institute of Biological Sciences, Federal University of Juiz de Fora, Campus Universitário, Juiz de Fora 36036-330, Brazil; E-Mail: Antonia.ribeiro@ufjf.edu.br

**Keywords:** *Vernonia polyanthes*, antinociceptive effect, anti-inflammatory effect

## Abstract

The ethanol extract from *Vernonia polyanthes* leaves (EEVP) was investigated for antinociceptive and anti-inflammatory effects at the doses (p.o.) of 100, 200 and 400 mg/kg in animal models. The extract reduced the number of abdominal contortions by 16.75% and 31.44% at a dose of 200 and 400 mg/kg, respectively. The results obtained showed that EEVP exerted a significant antinociceptive effect in the two phases of formalin. The EEVP increased the reaction time on a hot plate at the doses of 100, 200 and 400 mg/kg after 90 min of treatment. The paw edema was reduced by EEVP at the doses of 100, 200 and 400 mg/kg after 4 h of application of carrageenan. Doses of 200 and 400 mg/kg, administered 4 h before the carrageenan injection, significantly reduced the exudate volume (29.25 and 45.74%, respectively) and leukocyte migration (18.19 and 27.95%, respectively). These results suggest that *V. polyanthes* can be an active source of substances with antinociceptive and anti-inflammatory activities.

## 1. Introduction

Inflammation is a reaction of the body against an aggressive agent, characterized by vasodilatation and access of fluid and cells to the target tissue [1]. One of the major signs of inflammation is the pain that can be triggered by direct stimulation of nociceptors or by the action of inflammatory mediators [2]. These mediators, for example cytokines, histamine, serotonin, leukotrienes and prostaglandins, increase the vascular permeability and the migration of leukocytes to inflamed tissue [2]. In addition, different pathologic processes, such as cardiovascular and metabolic disorders [3–5], peptic ulcer [6] and cancer [7] are related to inflammation. The usual treatment of inflammatory pain is done by non-steroidal anti-inflammatory drugs, but the adverse effects described as irritation of gastric mucosa and ulcer, water retention and nephrotoxicity prevent the use of these agents [8]. On the other hand, medicinal plants have been widely used in traditional medicine to treat different inflammatory conditions [9]. However, the pharmacological properties and identifying the active compounds of these plants are needed to ensure its effectiveness and safety of users based on scientific support.

The genus *Vernonia*, one of the largest and most important member of the Asteraceae family, is represented by approximately 1500 species [10]. Plants of this genus, as *Vernonia polyanthes* Less, are found in South America, and have been traditionally used as diuretic, hypotensive, antihemorragic, sedative, abortive, anthelmintic, antiulcerogenic, antirheumatic, cicatrizing and anti-inflammatory [11,12]. Among different studies of *Vernonia*, the investigations regarding antinociceptive and anti-inflammatory effects are particularly important [13–15].

*V. polyanthes* Less (Asteraceae), known as assa-peixe, have been studied and their pharmacological properties such as antihypertensive and diuretic [12,16], antiulcerogenic [17], antifungal and leishmanicidal [18] and antimycobacterial [19] have been established. However, the antinociceptive and anti-inflammatory activities were not previously reported using this plant. Fixed acids, alkaloids, aminoacids, coumarins, steroids, triterpenes, anthraquinones, flavonoids, saponins and tannins were detected in infusions of *V. polyanthes* [20] and may be responsible for their pharmacological effects.

Considering the medicinal use for treatment of rheumatism, cicatrisation and inflammation and the lack scientific validation supported in pharmacological and clinical studies, the present investigation was designed to evaluate the antinociceptive and anti-inflammatory effects of EEVP using experimental animal models. In addition, preliminary phytochemical screening was conducted in order to determine the presence of the main classes of constituents in this extract.

## 2. Results and Discussion

### 2.1. Acute Toxicity

At the doses administered per oral route (p.o.), the EEVP was toxic to animals with LD_50_ of 2.78 g/kg (95% confidence intervals 1.67–4.64 g/kg). However, in the evaluated period, the animals did not show cyanosis, piloerection, writhing, ptosis, tremors, convulsions, ataxia, hypnosis, red urine or diarrhea. There were not alteration when considered the parameters motor activity, respiration, corneal reflex, righting and withdrawal, body tone and amount of pats.

### 2.2. Acetic Acid-Induced Writhing Response in Mice

The treatment of animals with EEVP (200 and 400 mg/kg, p.o.) produced a significant (*p* < 0.01 and *p* < 0.001, respectively) and dose-dependent inhibition in abdominal writhes produced by acetic acid (Table 1). The control group produced 66.37 ± 2.19 abdominal contortions.

### 2.3. Formalin-Induced Paw Licking in Mice

**Figure 1:**
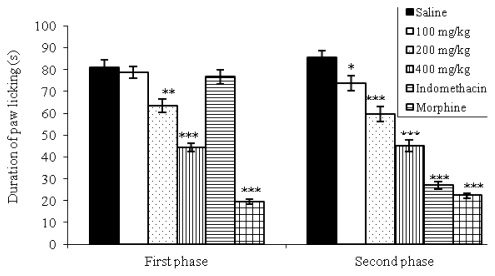
Effects of the EEVP on formalin-induced nociception in mice. First phase = 0–5 min after formalin injection; second phase = 15–30 min. Data are mean ± S.E.M. of eight mice. ** *p* < 0.01; *** *p* < 0.001 *vs.* control group.

In Figure 1, it is shown that pretreatment with morphine (5 mg/kg) or with the extract of *V. polyanthes* (200 and 400 mg/kg, p.o.) produced significant changes of paw licking time in the first phase of pain response. In the second phase, a dose-dependent and significant (*p* < 0.05 or *p* < 0.001) reduction in licking time was observed in mice treated with EEVP (100, 200 and 400 mg/kg, p.o.) as well as with indomethacin (10 mg/kg, p.o.) and morphine (5 mg/kg, s.c.). For the control group, the time spent was 80.87 ± 3.47 s and 85.50 ± 3.19 s in the first and second phases, respectively.

### 2.4. Effects on Hot-Plate Latency Assay in Mice

**Figure 2:**
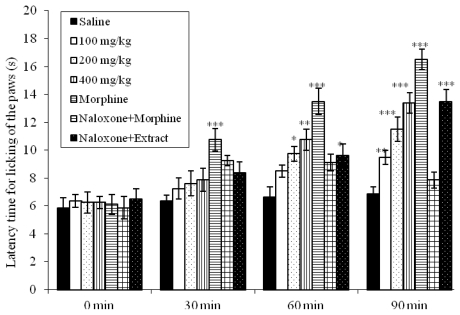
Effects of the EEVP on the latency time of mice exposed to the hot plate test. Data are mean ± S.E.M. of eight mice. * *p* < 0.05, ** *p* < 0.01, *** *p* < 0.001 *vs*. control group.

Due to the analgesic effect observed in the first phase of formalin test, we decided to evaluate the EEVP using hot plate test, a model of central antinociceptive activity. After 90 min of treatment, doses of 100 (*p* < 0.01), 200 (*p* < 0.001) and 400 mg/kg (*p* < 0.001) increased significantly the latency time in the respective control group (Figure 2). Morphine proved to be a potent analgesic, increasing the latency time within the evaluation periods. Naloxone, an opioid antagonist, blocked the morphine action but did not alter the antinociceptive effect of the EEVP.

### 2.5. Effects on Carrageenan-Induced Edema in Rats

The activity of the EEVP on carrageenan-induced edema in rat is shown in Table 2. After 2 h of carrageenan application, the paw edema was reduced in 16.95 and 22.03% at the doses of 200 and 400 mg/kg, respectively. Edema inhibition was observed 3 and 4 h after injection of carrageenan at the doses of 100 mg/kg (12.50 and 16.40%; *p* < 0.05, respectively), 200 mg/kg (16.41 and 27.87%; *p* < 0.05 and *p* < 0.01, respectively) and 400 mg/kg (25.78 and 31.97%; *p* < 0.01 and *p* < 0.001, respectively) when compared with control group. In these times, indomethacin (reference drug) also inhibited the paw edema (28.12 and 36.06%, respectively).

### 2.6. Effects on Carrageenan-Induced Pleurisy in Rats

The pleurisy effects demonstrated that doses of 200 (*p* < 0.05) and 400 mg/kg (*p* < 0.001) of EEVP significantly reduced the exudate volume in 13.83 and 43.08% (Table 3) when compared with control group. The number of total leukocytes was also inhibited at the doses of 200 (17.87%; *p* < 0.001) and 400 mg/kg (28.39%; *p* < 0.001) (Table 3) in comparison to the respective control. Indomethacin reduced the exudate volume and the leukocyte migration.

### 2.7. Phytochemical Screening

Phytochemical screening of the ethanol extract indicated the presence of flavonoids, tannins, coumarins, terpenoids, sterols, saponins and alkaloids.

The present study assessed the pharmacological effects of the leaves of *V. polyanthes*, a medicinal plant reputed in Brazilian folk medicine for its anti-inflammatory and cicatrizing properties [11,12]. Based on popular reports, the EEVP was prepared and screened for their antinociceptive and anti-inflammatory effects using classical models of nociception in mice and inflammation in rats. Therefore, considering the results of pharmacological tests observed in the present investigation, the EEVP has antinociceptive and anti-inflammatory effects and this is the first report described in the literature.

The acute toxicity test showed that the doses of EEVP were toxic to mice. Based in this result, the pharmacological dose (400 mg/kg maximal dose), not described previously in the literature, was defined from the LD_50_. The toxic effects have been described on plants of the genus *Vernonia* [13,14,21–24]. It is possible that the toxic effect of the EEVP could be due to the presence of phytochemical compounds as saponins detected in this study [24,25].

The writhing test is an experimental model used for the screening of drugs with analgesic activity, based on the irritation caused after intraperitoneal injection of 0.6% acetic acid. The writhing response is considered to be a visceral inflammatory pain model [26], and in this way, this acid causes the release of pain mediators such as bradykinin, prostaglandins, histamine and serotonin in the peritoneal fluid of mice [27]. In the present study, we clearly showed a dose-related antinociceptive effect of EEVP in the writhing test and probably this action could be due the presence of bioactive substances. This action could be mediated by peripherical effects, including the prostaglandin synthesis inhibition.

According to the Figure 1, the EEVP produced significant inhibition in the both phases of formalin-induced pain from the 200 mg/kg. The formalin test is a valid and reliable model of nociception and is sensitive for various classes of analgesic drugs. This test produced a distinct biphasic response and different analgesics may act differentially in the first and second phases [28]. It is considered a model to clinical pain because it causes a local tissue injury to the paw and is also indicative of tonic and localized inflammation pain. Moreover, this model can be used to clarify the possible mechanism of antinociceptive effect of a proposed analgesic [29]. Centrally acting drugs such as opioids inhibit both phases equally [30]. But peripherally acting drugs such as aspirin, indomethacin and dexamethasone only inhibit the late phase. The second phase seems to be an inflammatory response with inflammatory pain that can be inhibited by anti-inflammatory drugs [28,31]. Substance P and bradykinin act as mediators in the first phase, while histamine, serotonin, prostaglandin and bradykinin are involved in the nociceptive response of the second stage [30].

In the hot plate test, a central model that has a selectivity for opioid-derived analgesics [32], oral treatment with EEVP exerts an antinociceptive action confirming the central activity observed in the first phase of formalin test (100, 200 and 400 mg/kg). This test is also considered to be sensitive to drugs acting at the supraspinal modulation level of the pain response [33], suggesting at least a modulatory effect of the investigated extract. Our results indicate that the analgesia induced by the EEVP is not dependent on the opioid system, since previous treatment with naloxone did not change the observed data (Figure 2).

Carrageenan induced paw edema is an experimental model of acute inflammation involving different phases [34]. The first phase (1–2 h) is related with the release of serotonin and histamine; kinins play a role in the middle phase [33], while prostaglandins appear to be the most important mediators in the second phase (3–5 h) of the postcarrageenan response [35–37]. Based on this explanation, it could be argued that the suppression of the first phase may be due to inhibition of the release of early mediators, such as histamine and serotonin, and the action in the second phase may be explained by an inhibition of cyclooxygenase. Therefore, the present result indicates that ethanol extract (100, 200 and 400 mg/kg, p.o.) and indomethacin play a crucial role as protective factors against the carrageenan-induced acute inflammation.

The injection of carrageenan into the pleural cavity of rats elicited an acute inflammatory response, characterized by the accumulation of fluid containing large number of leukocytes [38–40]. It is an interesting method that evaluates the leukocyte migration during the inflammatory process. Non-steroidal anti-inflammatory drugs, such as indomethacin, inhibit the accumulation of exudates and mobilization of leukocytes between 3 and 6 h after application of carrageenan [39,41]. In our experiment, the EEVP in the pleurisy model clearly showed the inhibition of the formation of pleural exudate and the leukocyte migration (Table 3).

Studies have been reported the antinociceptive and anti-inflammatory effects of plants of the genus *Vernonia* [13–15]. Among the active compounds identified in this important genus, the highlights are the flavonoids [42–44]. In our investigation, phytochemical screening showed the presence of numerous constituents’ classes, such as flavonoids, tannins, coumarins, terpenoids, sterols, saponins and alkaloids. Considering this chemical diversity, pharmacological effects could be attributed to the tannins and flavonoids, since these constituents are well established as antinociceptive and/or anti-inflammatory agents [45–47]. However, complementary studies are necessary to determine the better correlation between activities and chemical composition of *V. polyanthes*.

## 3. Experimental Section

### 3.1. Plant Material and Extraction

The plant material used in the present study was collected in Juiz de Fora, Minas Gerais State, Southeast region of Brazil, in March 2009. The species was identified by Dr. Fátima Regina Gonçalves Salimena and a voucher specimen (CESJ number 10.329) was deposited in the Herbarium of the Federal University of Juiz de Fora, Brazil. Dried and powdered leaves (450 g) were exhaustively extracted in 95% ethanol (2.5 L) by static maceration for 3 weeks at room temperature with renewal of solvent every 2 days. The EEVP was filtered and evaporated under a rotary evaporator at controlled temperature (50–60 °C). This material was placed in a desiccator with silica to yield 36.68 g. The dried extract was dissolved using 1% DMSO in normal saline for pharmacological studies.

### 3.2. Chemicals

Drugs and reagents used in this study (and their sources) were as follows: acetic acid and acetylsalicylic acid (Vetec Química Farm Ltda, Rio de Janeiro, RJ, Brazil), formaldehyde (Reagen Quimibrás Ind. Química S.A., Rio de Janeiro, RJ, Brazil), morphine hydrochloride (Merck Inc., Whitehouse Station, NJ, USA), naloxone and indomethacin (Sigma Chemical Co, St Louis, MI, USA).

### 3.3. Animals

Male Wistar rats (90–110 days) weighing 200–240 g and male Swiss albino mice (50–70 days) weighing 25–30 g were used in the experiments. The animals were provided by the Central Biotery of the Federal University of Juiz de Fora. The animals were divided into groups and kept in plastic cages (47 × 34 × 18 cm) under a 12 h light/12 h dark cycle at room temperature (22 ± 2 °C), with free access to Purina rations and water. Animal care and the experimental protocol followed the principles and guidelines suggested by the Brazilian College of Animal Experimentation (COBEA) and were approved by the local ethical committee (protocol number 053/2009).

### 3.4. Acute Toxicity

Groups of ten mice received oral doses of 0.5, 1, 1.5, 2 and 3 g/kg of EEVP, while the control group received the vehicle (saline). The groups were observed for 48 h and mortality at end of this period was recorded for each group [48]. The LD_50_ (50% lethal dose) was determined by probit test using the log of the dose *versus* probit [49]. The determination of LD_50_ served to define the doses used in the experiments of pharmacological activities.

### 3.5. Writhing Test

Antinociceptive activity was evaluated on the acetic acid-induced writhing according to Collier *et al*. protocol [26]. Male Swiss albino mice were divided into groups of eight mice. The animals were pretreated with EEVP (100, 200, and 400 mg/kg, p.o.) or acetylsalicylic acid (200 mg/kg, p.o.) and indomethacin (10 mg/kg, p.o.) used as standard drugs, one hour prior to intraperitoneal (i.p.) of 0.6% v/v acetic acid (0.1 mL/10 g). Ten minutes after i.p. injection of acetic acid, the number of writhing during the following 20 min were counted. Control mice received 1% DMSO in sterile saline orally (10 mL/Kg).

### 3.6. Formalin Test

The method previously described by Hunskaar and Hole [28] was used. Pain was induced by injecting of 20 μL of 2.5% formalin (37% formaldehyde) in sterile saline in the subplantar right hind paw region. Male mice were divided into groups of eight mice each. Extract was administered orally at doses of 100, 200, and 400 mg/kg body wt., 60 min before formalin injection. The control group received 1% DMSO in sterile saline orally (10 mL/kg), while indomethacin (10 mg/kg, p.o.) and morphine (5 mg/kg subcutaneous) were used as positive controls. The animals were observed to evaluate the licking time (an index of nociception) during the first phase, neurogenic (0–5 min), and the second phase, inflammatory (15–30 min), after formalin injection.

### 3.7. Hot-Plate Test

The hot plate test was carried out according to the method described by Eddy and Leimbach [50]. Mice were placed on a hot plate (Model LE 7406, Letica Scientific Instruments, Barcelona, Spain) maintained at 55 ± 1 °C. Latency of nociceptive response such as licking, flicking of a hind limb or jumping was measured. Three groups of mice (*n* = 8) were treated p.o. with EEVP (100, 200 or 400 mg/kg; 0.1 mL per 10 g body weight); the control group received 1% DMSO in sterile saline (10 mL/kg). Measurements were performed at time 0, 30, 60 and 90 min after drug administration, with a cut-off time of 40 s to avoid lesions to the animals’ paws. The effect of pretreatment with naloxone (1 mg/kg, subcutaneously) on the analgesia produced by the EEVP (400 mg/kg) was determined in a separate group of animals. Morphine (5 mg/kg, subcutaneously), in the absence and presence of naloxone treatment, was used as a reference.

### 3.8. Carrageenan-Induced Paw Edema

According to the method described by Winter *et al*. [51], the paw edema volume of the rats was measured using a plethysmometer (model LE 7500, Letica Scientific Instruments, Barcelona, Spain). Male Wistar rats were divided into groups of six animals which received, p.o., doses of EEVP (100, 200 and 400 mg/kg; 0.1 mL per 10 g body weight), 1% DMSO in saline or indomethacin (10 mg/kg) 1 h before the injection of carrageenan. The animals were anesthetized with cetamine and xylazine (60 mg/kg and 8 mg/kg, respectively) 30 min before the injection of 0.1 mL of 2% (w/v) carrageenan into the subplantar region of the right hind paw for the induction of edema. In the left hind paw, used as a control, 0.1 mL of sterile saline was injected. The volume of paw edema, recorded three times, was measured at 1, 2, 3 and 4 h after carrageenan injection. The measure of edema was made by the difference between the volume displaced by the right paw and the left paw.

### 3.9. Carrageenan-Induced Pleurisy

Rats were anesthetized with cetamine and xylazine (60 mg/kg and 8 mg/kg, respectively) and a suspension of saline containing 2% carrageenan (0.4 mL) were injected into the pleural cavity [41]. EEVP (100, 200 and 400 mg/kg), 1% DMSO in saline or indomethacin (10 mg/kg, p.o.) were given 60 min before injection of the irritant. At 4 h after the injection of carrageenan, the animals were killed by overdose of cetamine and xylazine (120 mg/kg and 16 mg/kg, respectively), and the skin and pectoral muscles were retracted. A longitudinal incision was made between the third and fifth ribs on each side of the mediastinum. The exudate was collected and transferred to a 15 mL conical centrifuge tube and the total volume determined. A 20 μL aliquot of the exudate was used to determine the total leukocyte count in Neubauer chambers.

### 3.10. Phytochemical Screening of the EEVP

Phytochemical screening of the EEVP was performed to detect the eventual presence of different classes of constituents, such as alkaloids, flavonoids, anthraquinones, coumarins, saponins, terpenes and tannins using specific reactions [52].

### 3.11. Statistical Analysis

Data are expressed as mean ± S.E.M. Statistical significance was determined by one-way analysis of variance followed by the Student–Newman–Keuls test. *P* values below 0.05 were considered significant. The percentage of inhibition was calculated by using

100-T×100/C (%) or T×100/C-100 (%)

where C and T indicate non-treated (vehicle) and drug-treated, respectively.

## 4. Conclusions

Our results clearly demonstrated that the EEVP displayed significant antinociceptive and anti-inflammatory effects in mice and rats models. In addition, these results provided an initial scientific validation of popular use of this plant as a medicine against dolorous and inflammatory processes.

## Figures and Tables

**Table 1:** Effects of the ethanol extract from *Vernonia polyanthes* leaves (EEVP) on acetic acid-induced writhing in mice.

Group	Dose (mg/kg)	Number of writhes	Inhibition (%)
Control	Saline	66.37 ± 2.19	-

	100	64.62 ± 2.44	2.64
EEVP	200	55.25 ± 2.39 **	16.75
	400	45.50 ± 2.44 ***	31.44

Indomethacin	10	22.62 ± 1.91 ***	65.92

Acetylsalicylic acid	200	20.62 ± 1.69 ***	68.93

Data are mean ± S.E.M. of eight mice.

** *p* < 0.01,

*** *p* < 0.001 *vs*. control group.

**Table 2:** Effects of the EEVP on carrageenan-induced paw edema in rats.

Group	Dose (mg/kg)	Volume of hind paw (mL)			

		1 h	2 h	3 h	4 h
Control	Saline	1.05 ± 0.08	1.18 ± 0.08	1.28 ± 0.06	1.22 ± 0.06

	100	0.97 ± 0.06	1.03 ± 0.04	1.12 ± 0.05 *	1.02 ± 0.08 *
EEVP	200	0.92 ± 0.06	0.98 ± 0.05 *	1.07 ± 0.04 *	0.88 ± 0.04 **
	400	0.85 ± 0.04	0.92 ± 0.05 *	0.95 ± 0.04 ***	0.83 ± 0.04 ***
Indomethacin	10	0.82 ± 0.05	0.85 ± 0.06 **	0.92 ± 0.05 ***	0.78 ± 0.04 ***

Data are mean ± S.E.M. of six rats.

* *p* < 0.05,

** *p* < 0.01,

*** *p* < 0.001 *vs*. control group.

**Table 3:** Effects of the EEVP on number of leukocytes in carrageenan-induced pleurisy in rats.

Group	Dose (mg/kg)	Exsudate volume (mL)	Inhibition (%)	N° Leukocytes (10^3^ cells/mm^3^)	Inhibiti on (%)
Control	Saline	1.88 ± 0.08	-	15.50 ± 0.50	-

	100	1.78 ± 0.08	5.32	14.90 ± 0.43	3.87
EEVP	200	1.62 ± 0.04 *	13.83	12.73 ± 0.21 ***	17.87
	400	1.07 ± 0.05 ***	43.08	11.10 ± 0.32 ***	28.39

Indomethacin	10	0.73 ± 0.09 ***	61.17	9.87 ± 0.35 ***	36.32

Data are mean ± S.E.M. of six rats.

* *p* < 0.05,

*** *p* < 0.001 *vs*. control group.
